# Unveiling the effect of strain engineering on the electrochemical properties of hydrothermally grown nanostructured indium doped ZnSeO_3_ for photoanode applications

**DOI:** 10.1038/s41598-023-47436-7

**Published:** 2023-11-16

**Authors:** M. W. Maswanganye, G. L. Kabongo, L. E. Mathevula, B. M. Mothudi, M. S. Dhlamini

**Affiliations:** 1 Department of Physics, University of South Africa, Florida Park, Roodepoort, 1709 Republic of South Africa; 2 Department of Physics, University of Limpopo, Private Bag X1106, Sovenga, 0727 South Africa

**Keywords:** Energy science and technology, Materials science, Nanoscience and technology

## Abstract

The crucial role of In as a dopant on the structural, optical, and thermogravimetric characteristics of the zinc selenite (ZnSeO_3_) nanopowders has been investigated in detail using X-Ray diffraction (XRD), field emission scanning electron microscopy (FE-SEM), Energy Dispersive Spectroscopy (EDS), Raman spectroscopy, diffuse reflectance spectroscopy (DRS), photoluminescence (PL) spectroscopy, and Thermogravimetric Analysis (TGA). The structural analysis indicates that all patterns are assigned to the ZnSeO_3_ orthorhombic structure. Also, XRD analysis shows that In^3+^ ions may have replaced Zn^2+^ ions, which causes lattice expansion. Both the Debye–Scherrer method, and the Williamson–Hall method have also been applied to study the influence of strain on the calculation of the crystallite size. The crystallite size was observed to increase with an increase in dopant concentration. The FE-SEM corroborated that the prepared samples are orthorhombic, with the EDS and mapping confirming the presence of In as a dopant. Raman spectroscopy results corroborated the XRD results indicating an expansion in the crystal structure of ZnSeO_3_ with the introduction of dopants. Based on DRS data, the introduction of In decreases the energy band gap of the synthesized ZnSeO_3_ nanopowder samples from 3.305 to 3.276. PL spectra confirm the presence of indium with the green emission band attributed to dopants dominating the emission. The TGA investigation shows an improvement in the mass loss with the introduction of dopants. EIS results indicated an improvement in the conductivity as the charge transfer resistance decreased from 525.04 to 21.95 kΩ for the undoped ZnSeO_3_ and 0.75% In–ZnSeO_3_ thin films showing improvement in charge mobility.

## Introduction

The migration to the fourth industrial revolution will be successful with a variety of functional and sustainable renewable energy infrastructure. The generation, storage, and inversion of electricity is regarded by scientists around the world as one of the most pressing issues that need an immediate attention. Dye-sensitized solar cells (DSSCs), quantum-dot solar cells, and perovskite-based solar cells are among the technologies of the third generation of solar cells that can be used to heaviest electricity as the sun is in abundance. Due to their low cost, high performance, and ease of production, DSSCs have emerged as an effective technology among these technologies. A typical DSSC consists of a sensitizer (dye), working electrode, counter electrode, electrolyte, and a transparent conducting such as ITO (indium-doped tin oxide) or FTO (fluorine-doped tin oxide) layer^1,2^. However, there is a setback in the development of this research field as the photoanode materials have poor performance^3^. Photoanode is the most significant component of DSSCs, and its photo conversion efficiency (PCE) is dictated mostly by other characteristics such as photo generated charge carrier recombination and carrier mobility. An excellent photoanode must have a distinct nanostructure morphology with a low band gap, an appro-priate surface area, and adequate porosity.

By altering the physical properties of these materials, the PCE of DSSCs can be improved. Doping, surface modification and the use of semiconducting composites have all been proposed as means to improve photoanode PCE^4–6f^. Physical properties in the majority of materials can be tuned over by precisely manipulating their structures (for example bond length, angle, and relative positions of atoms)^7^.The strain is the change of the positions of atoms, or the lengths of the synthetic bonds inside a crystal that are prompted by the application of stress which causes defects into the crystal structure of a material. As a result of the presence of lattice defects in bulk three-dimensional materials, the low disappointment strains of bulk crystalline materials limit the degree to which their properties can be changed^7,8^. However, this can be made possible by introducing dopants or by bringing different materials together. Different researchers have combined materials such as CdO and TiO_2_^9^, ZnO and ZnSe in order to create nanostructured zinc selenite (ZnSeO_3_) for photoanodes, photo catalysis, catalysis and different possible applications^10^.There are still a lot of properties that are not known about ZnSeO_3_ and this is a disadvantage in applications such as DSSCs. Incorporation of supporting material such as dopant into the ZnSeO_3_ is a smart way to explore and improve the properties of ZnO and ZnSe together instead of using them individually^10^.

Zinc oxide (ZnO) is among the most generally utilized n-type metal oxide semiconductor materials. This is due to its exceptional structural, optical, and electrical properties in formation with cheap, nontoxic nature, and natural abundance^11^. It has an energy band gap of 3.37 eV, high electron mobility and a large exciton binding energy of 60 meV which might capably produce photon emission in the ultraviolet range at room temperature^12,13^. These properties makes ZnO to be applicable in DSSCs solar cells^14,15^. Another material of interest is Zinc Selenide (ZnSe) a material that also belongs to a clan of semiconductors. It is optically dynamic in the visible region and it is a promising material in optoelectronic devices like LEDs (blue laser diodes), photo detectors and photovoltaic devices because of its direct band gap (2.67 eV) and enormous bonding energy exciton (21 meV) at room temperature^16^. ZnSe has a smaller band gap—compared to ZnO, however, its valence and conduction bands are accustomed slightly to those of ZnO indicating that the combination of the two metal oxides can bring about improvement for nanostructured materials in applications such as photovoltaic^17^. Different methods such as Electro deposition^18^, Pulse Laser Deposition (PLD)^19^, hydrothermal^10^ have been employed to synthesize nano-structured ZnSeO_3_. Hydrothermal is essentially a solution-reaction technique. The preparation of nanomaterials in hydrothermal synthesis can occur at temperatures ranging from room temperature to extremely high temperatures, either at low-pressure or high-pressure conditions and it can be utilized to control the morphology of the materials^20^.

In the present work, undoped ZnSeO_3_ and Indium doped ZnSeO_3_ have been successfully synthesised via hydrothermal method and strain analysis was conducted to elucidate the effect of strain induced in ZnSeO_3_ via doping. Finally, electrochemical Impedance Spectroscopy analysis of the fabricated films revealed the dependence of charge mobility on strain, this is a promising finding that can lead to improving photoanode charge mobility properties for DSSC applications.

## Experimental details

### Sample preparation

The undoped-ZnSeO_3_ nanopowders and Indium doped ZnSeO_3_nanopowders were synthesized by hydrothermal method. Zinc acetate dehydrate [Zn(CH_3_CO_2_)_2_·2H_2_O], Sodium selenite[Na_2_Se_3_O_3_], Indium acetate anhydrous [In(CH_3_COO)_3_] (all purity of 98.0% from Sigma-Aldrich) were used as precursor materials. Deionized water was used as the only solvent. 

**Figure 1:**
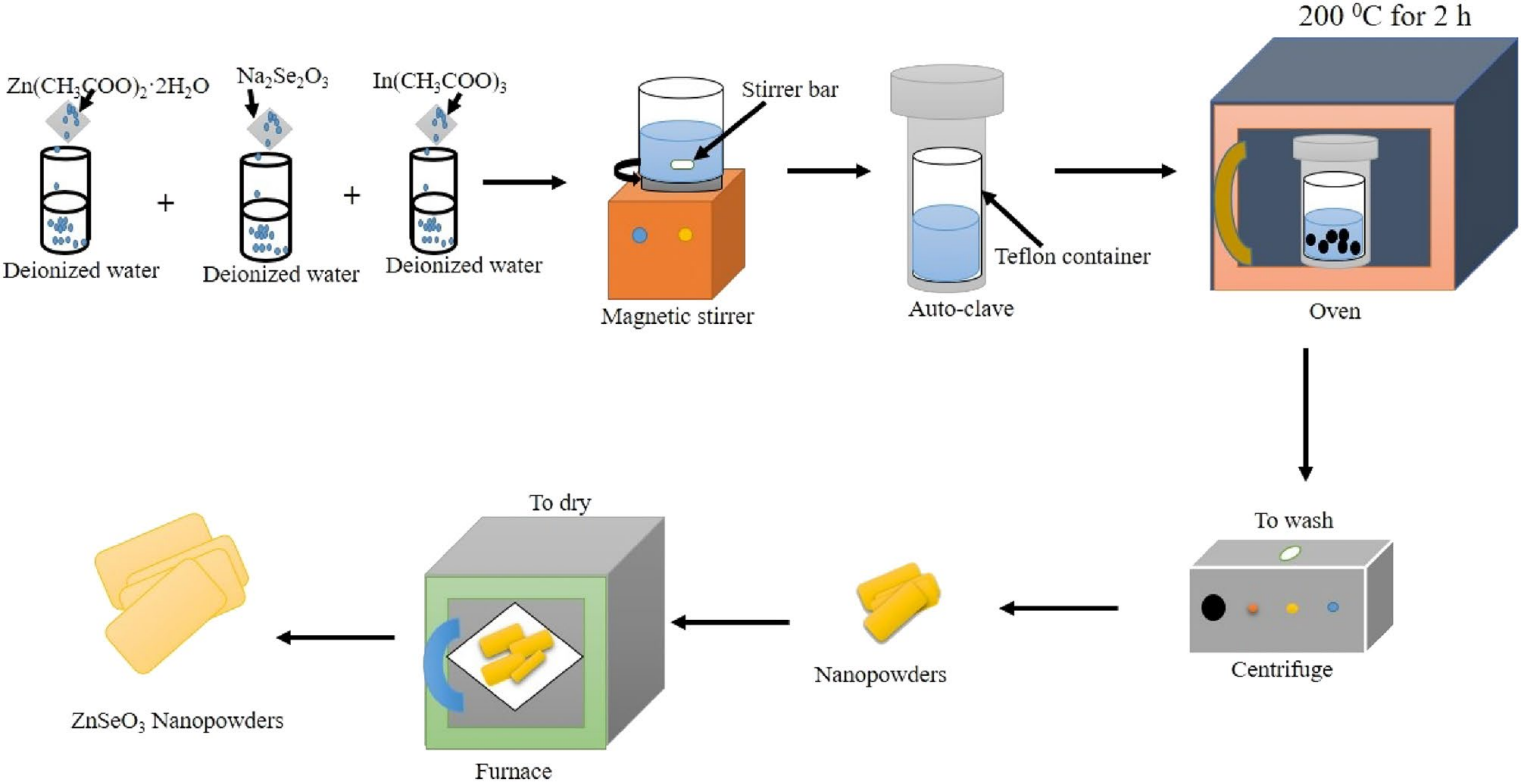
Schematic synthesis method for ZnSeO_3_nanopowder samples.

For the preparation of the undoped-ZnSeO_3_ nanopowder sample, 0.005 mol of Zn(CH_3_CO_2_)_2_·2H_2_O and 0.005 mol of Na_2_Se_3_O_3_ were dissolved and stirred in 10 mL deionized water for 10 min as shown in Fig. 1. After continuous magnetic stirring for 10 min, solutions were mixed and stirred for another 10 min. For the preparation of In–ZnSeO_3_ nanopowder samples, 0.25 mol%, 0.50 mol%, and 0.75 mol%, of In(CH_3_COO)_3_ solutions were dissolved and stirred in 10 mL deionized water for 10 min. After continuous magnetic stirring for 10 min, solution were mixed together stirred for 10 min. The final solutions for both undoped and In-doped ZnSeO_3_ samples were then poured into a Teflon then placed into stainless-steel auto-claves. Then the stainless-steel auto-claves were placed inside an oven for 2 h at 200 ℃. The stainless-steel auto-claves were later allowed to cool down naturally at room temperature where the products were collected and washed in a centrifuge with deionized water five times at rpm 4000 for 10 min. After washing, the samples were then dried at 60 ℃ overnight. An illustration representing the process followed is shown in Fig. 1.

### Characterization

A Rigaku Smart Lab diffractometer (Cu-K radiation, = 1.5406 Å) was used for producing the X-ray diffraction (XRD) patterns. JEOL JSM-7800F field emission scanning electron microscopy (FE-SEM) was used to assess the morphology. A 532 nm laser was used to perform Raman measurements with a Horiba Xplora PLUS Raman Microscope. The materials’ diffuse reflectance properties were investigated using a Perkin Elmer Lambda 1050 UV–Vis-NIR spectrophotometer. At room temperature, the measurements were taken in reflection mode using the reduced reflectance technique. The emission spectra of room temperature photoluminescence (PL) were measured using a Fluorolog Horiba Jobin–Yvon equipped with a 450W xenon lamp as an excitation source. Thermogravimetric Analysis (TGA) was performed on the Q600 SDT TGA analyzer.

A 1 M electrolyte solution of NaSO_4_ was used for the measurements in the electrochemical impedance spectroscopy (EIS). The nanopowders of undoped ZnSeO3 and 0.75% In–ZnSeO3 were mixed with polyvinylidene difluoride. (PVDF). Later, a few drops of DMSO were added to the mixture to form a precipitate. After that, the precipitate was sonicated for 30 min. The precipitate was then drop coated onto a 2.5 cm × 1.8 cm ITO conductive glass substrate. The glass substrates were then dried in an oven at 60 degrees Celsius for 60 minutes^21^. A reference electrode of Ag/AgCl chloride was used, and a counter electrode of Pt was connected to an Autolab instrument. At an 8 cm distance, the working electrode was placed upright, with the base part facing the source of light (solar simulator with a xenon lamp produces 100 mW cm^-2^ visible light intensity)^21^. The EIS measurements were performed at frequencies ranging from 0.1 Hz to 100 kHz, with an amplitude of 10 mV.

## Results and discussions

### X-ray diffraction

**Figure 2:**
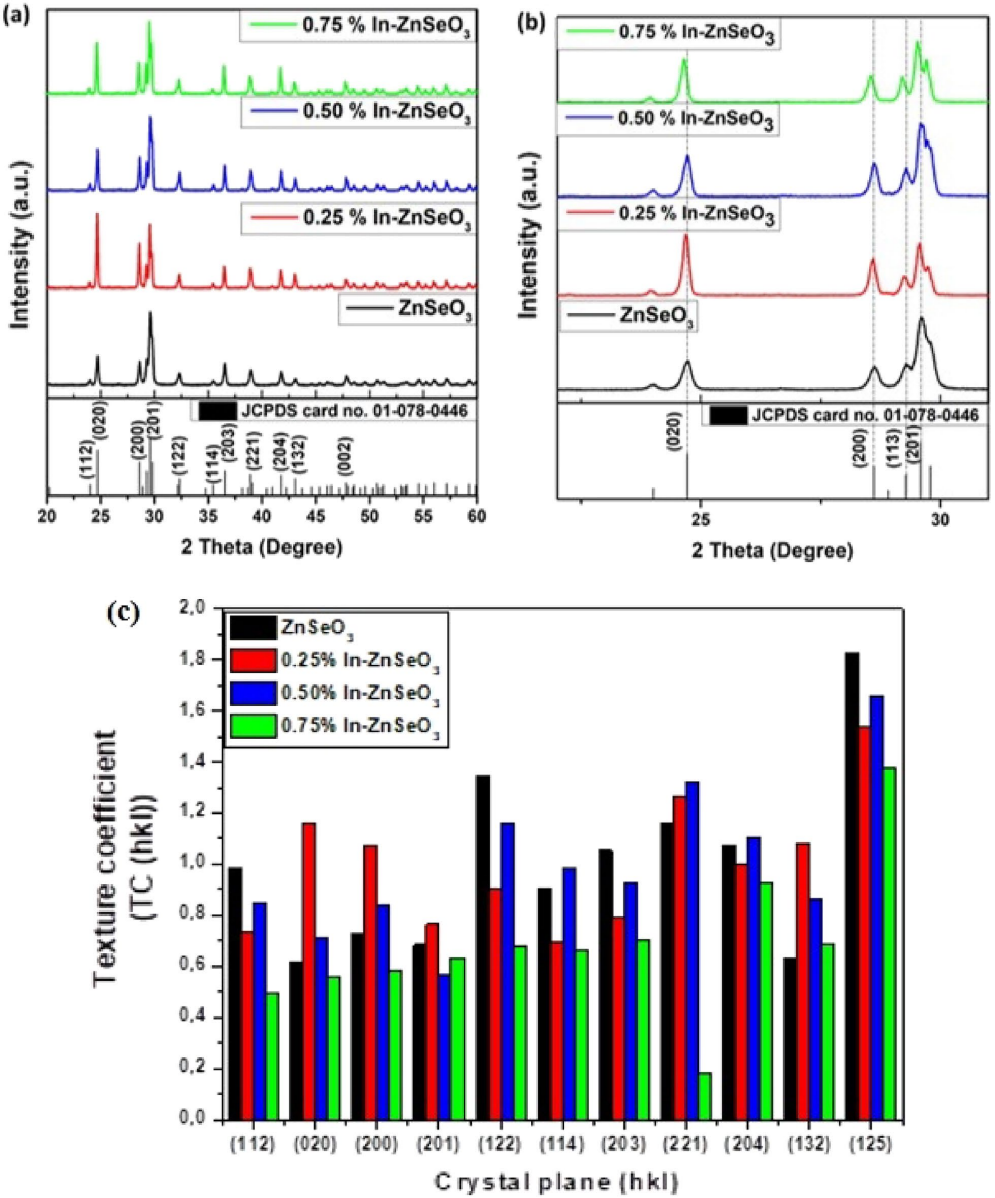
(**a**)XRD patterns of undoped ZnSeO_3_and In-doped ZnSeO_3_ nanopowdersamples, (**b**) magnified region of the diffraction patterns showing peak shift at (020), (200) (113) (201), (**c**) Texture coefficient (TC (hkl)).

Crystal structures and phase analysis was investigated using X-ray diffraction (XRD). The analysis has been used to elucidate the crystal structure as well as lattice strain, as it is a clear way to study the lattice section strain. Figure 2a. Shows the XRD patterns of the undoped ZnSeO_3_ and In-doped ZnSeO_3_ nanopowder samples at different concentrations. All the prepared samples were found to be of ZnSeO_3_ orthorhombic structure (JCPDS No.01-078-0446), as shown in Fig. 2a. There were no impurities observed for the undoped ZnSeO_3_ nanopowder samples and also there were no In peaks observed for all the In-doped ZnSeO_3_ indicating that In was successfully doped into ZnSeO_3_. A peak shift towards the lower 2-theta angles has been observed with an increase in In concentration as indicated in Fig. 2b, a close examination of the XRD patterns uncovers that all the XRD peaks shift towards lower angles which indicates the presence of In in the ZnSeO_3_ crystal structure. The shifting towards lower angles shows that there is a lattice expansion in the In-dopedZnSeO_3_ nanopowder samples. That is because the incorporation of another element with a different lattice constant results in outcomes that cause a shift towards higher or lower angles due to lattice constriction or expansion, respectively^22^.

The lattice parameters a ≠ b ≠ c for the prepared ZnSeO_3_ nanopowder samples were determined using the following equations:

1 $$2{d}_{hkl}\mathrm{sin}\theta =n{\lambda }_{CuK\propto }$$

2 $$\frac{1}{{{\varvec{d}}}_{{\varvec{h}}{\varvec{k}}{\varvec{l}}}}=\frac{{{\varvec{h}}}^{2}}{{{\varvec{a}}}^{2}}+\frac{{{\varvec{k}}}^{2}}{{{\varvec{b}}}^{2}}+\frac{{{\varvec{l}}}^{2}}{{{\varvec{c}}}^{2}}$$

where ${\lambda }_{CuK\propto }$ is the wavelength of the XRD, n is the order of diffraction (for first order n = 1), d_hkl_ is the interplanar spacing and *hkl* are the Miller indices. The estimated lattice parameters a, b, and c of all the samples were found to be comparable with the reported values of the bulk ZnSeO_3_ (JCPDS No.01-078-0446). The values of the lattice parameters represented in Table 1 are observed to increase with an increase of In concentration. The volume of the unit cell of the prepared samples were also calculated using the following formula:

**Table 1:** Lattice parameters and unit cell volume of undoped ZnSeO_3_ and In-doped ZnSeO_3_ nanopowder samples.

Sample name	a (Å)	b (Å)	c (Å)	V (Å^3^)
ZnSeO_3_^23^	6.233	7.200	11.987	537.948
ZnSeO_3_	6.233	7.201	11.983	537.852
0.25% In–ZnSeO_3_	6.240	7.209	11.988	539.279
0.50% In–ZnSeO_3_	6.233	7.202	11.985	538.040
0.75% In–ZnSeO_3_	6.249	7.220	11.992	541.079

3 $$V=abc$$

The volumes of the unit cell for the prepared nanopowder samples were found to be increasing with an increase in In concentration, results are shown in Table 1. The increase indicates that In dopant might has substituted Zn in the crystal lattice with the larger ionic radii of In (80 pm) as compared to the ionic radii of Zn (74 pm) being the cause of the expansion.

Texture coefficients (TCs) are generally used to recognize the favoured orientation of growth along (hkl) direction. The favourable crystallite orientation of the examples can be determined as follows^24^:

4 $$TC\left({h}_{i}{k}_{i}{l}_{i}\right)=\frac{\frac{I\left({h}_{i}{k}_{i}{l}_{i}\right)}{{I}_{0}\left({h}_{i}{k}_{i}{l}_{i}\right)}}{\frac{1}{N}\sum_{j=1}^{N}\frac{I\left({h}_{j}{k}_{j}{l}_{j}\right)}{{I}_{0}\left({h}_{j}{k}_{j}{l}_{j}\right)}}$$

where I (hkl) is the obtained intensity of the prepared samples from the diffraction patterns, I_0_ (hkl) shows the reference intensity (JCPDS No.01-078-0446) and N is the quantity of diffraction peaks. On the off chance that the TC (hkl) is close to 1 for all (hkl) planes, then, at that point, the crystallite orientation of prepared nanopowder samples is equivalent to the JCPDS card. The TC values of ˃1 show the preferential development of the crystallite orientations in an exceptional (hkl) direction^24^. Figure 2c shows the TC curves of the prepared nanopowder samples. It is observed that the preferential growth of the prepared undoped-ZnSeO_3_ sample is situated along the (125) and (122) headings and slight texturing in the (221) direction. The In–ZnSeO_3_ samples are also observed to have (125) being the most preferential growth, with (221), (221) and (204) being the second preferred growth direction for the 0.25% In–ZnSeO_3_, 0.50% In–ZnSeO_3_, and 0.75% In–ZnSeO_3_ respectively.

The average crystallite size (D) and the micro strain (ε) of the prepared samples were determined from the XRD data using the Debye Scherrer equations^25^,

5 $$D=\frac{0.9{\lambda }_{CuK\propto }}{{\beta }_{hkl}\mathrm{cos}\theta }$$

6 $$\varepsilon =\frac{{\beta }_{hkl}\mathrm{cos}\theta }{4\mathrm{sin}\theta }$$

where λ_CuKα_ = 1.5406 Å, β_hkl_ is the full width at half maximum intensity (FWHM) of the XRD peaks in radians and θ is the Bragg’s angle of the diffraction peak. To ascertain the micro strain and average crystallite size of the synthesized nanopowder samples, the Williamson–Hall (W–H), Debye–Scherrer (D-S) and Size-strain Plot (SSP) methods were used. That is because W–H and SSP technique incorporates the values of the micro-strain in the calculation. The micro strain (Ɛ) and average crystallite size (D) from the W–H plot are determined by the accompanying Eq. ^24^:

7 $${\beta }_{hkl}\mathrm{cos}\theta =4\varepsilon \mathrm{sin}\theta +\frac{0.9{\lambda }_{CuK\propto }}{D}$$

**Figure 3:**
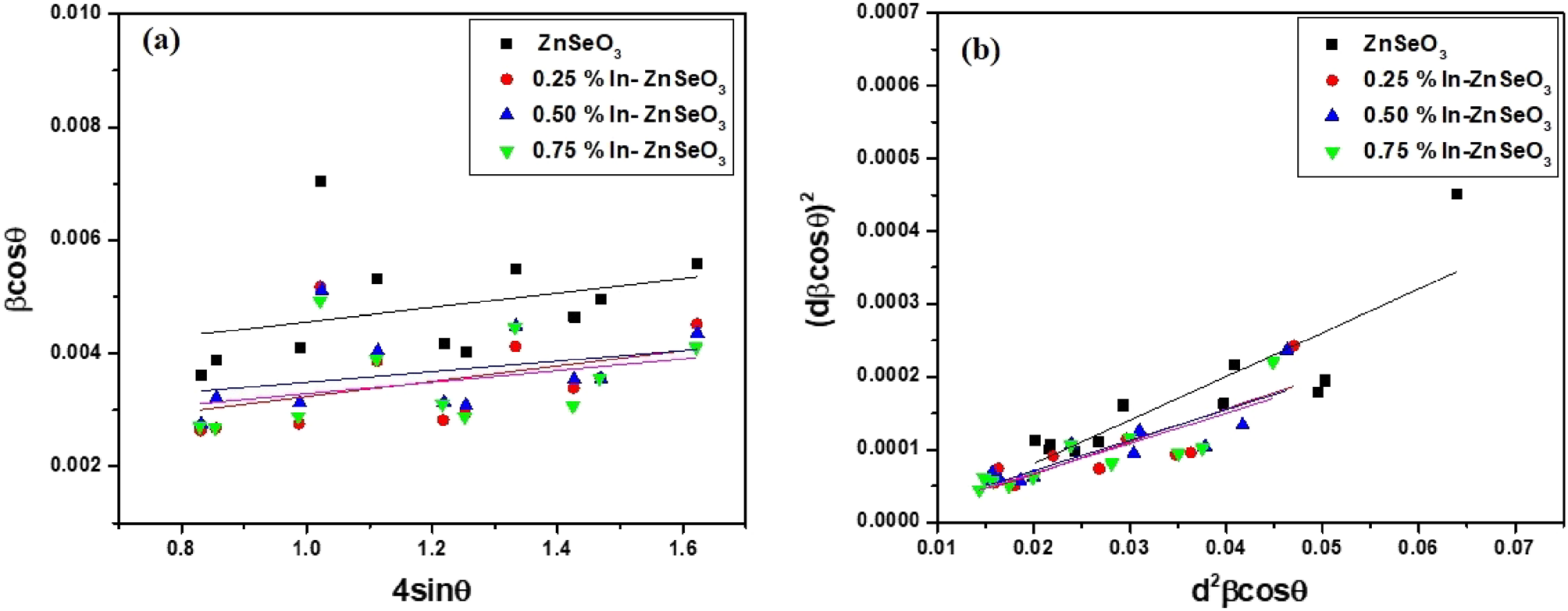
(**a**) Williamson–Hall plot (**b**) Size-strain plot of undoped ZnSeO_3_ and In-doped ZnSeO_3_nanopowder samples.

The plot of ${\beta }_{hkl}\mathrm{cos}\theta$ versus $4\mathrm{sin}\theta$ can give strain instigated on the particles and crystallite size which are proportional to the slope of the plot and y-intercept, respectively. While the SSP method was determined from the following Eq. ^26^:

8 $${\left({{d}_{hkl}\beta }_{hkl}\mathrm{cos}\theta \right)}^{2}={\left(\frac{\varepsilon }{2}\right)}^{2}+\frac{k}{D}{{{d}_{hkl}}^{2}\beta }_{hkl}\mathrm{cos}\theta $$

where k is a shape-dependent constant (3/4 for spherical particles). Figure 3 shows both W–H and SSP plots of the ZnO nanoparticle samples. The calculated values of the average crystallite sizes are shown in Table 2.

**Table 2:** The average calculated crystallite size and the micro strain (ε) of the synthesized samples.

Sample	D–S method		W–H method		SSP method	
	D (nm)	Ɛ (10^–3^)	D (nm)	Ɛ (10^–3^)	D (nm)	Ɛ (10^–3^)
ZnSeO_3_	29.95	4.14	42.14	1.26	12.48	12.59
0.25% In–ZnSeO_3_	41.62	3.00	73.75	1.36	16.70	9.76
0.50% In–ZnSeO_3_	39.04	3.17	54.16	0.93	17.86	7.29
0.75% In–ZnSeO_3_	41.46	3.00	61.35	1.03	18.16	7.84

The average crystallite sizes calculated using all the methods have been observed to increase with an increase of In concentration. The micro strains for the In–ZnSeO_3_ nanopowder samples determined using the W–H method were found to increase with an increase in average crystallite size, indicating that the doped samples are under tensile strain and not compressive strain. The values of the y-intercept determined using the SSP method were found to be negative also indicating that the samples are under tensile strain.

### Field emission scanning electron microscopy (FE-SEM) and energy dispersive spectroscopy (EDS)

**Figure 4:**
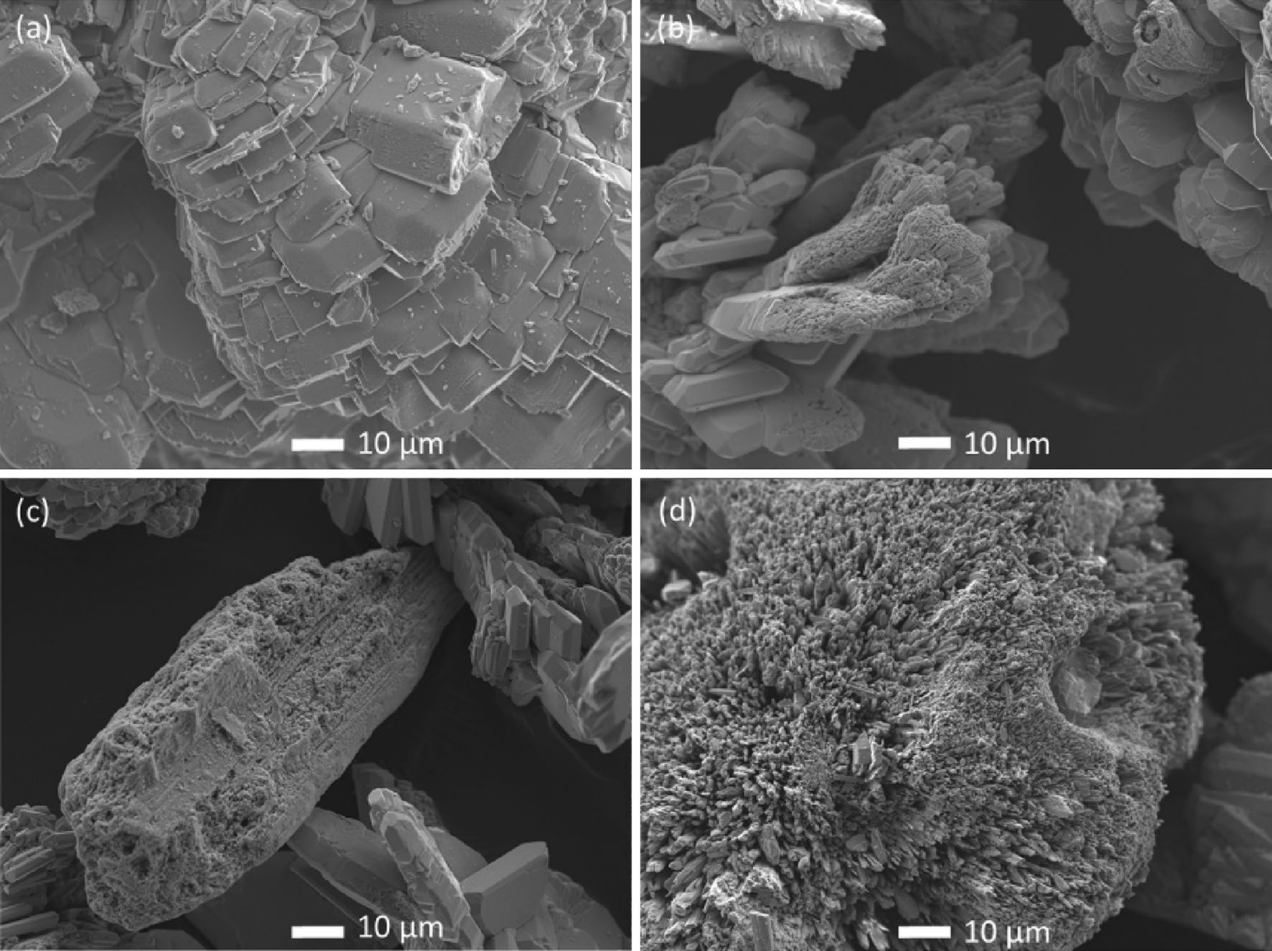
**FE-**SEM micrographs of (**a**) undoped ZnSeO_3_ and (**b**) 0.25%, (**c**) 0.50% and (**d**) 0.75% In-doped ZnSeO_3_nanopowder samples.

The investigation of surface morphology of the ZnSeO_3_ prepared samples was achieved by FE-SEM. The FE-SEM micrographs of the undoped-ZnSeO_3_ and In doped ZnSeO_3_ samples at different concentrations are displayed in Fig. 4. From Fig. 4, it is revealed that the shape of the prepared ZnSeO_3_ nanopowder samples are orthorhombic for all the samples and this corroborate the XRD results. From Fig. 4a it can be seen that the particles stack together with the introduction of dopants, changing the orientation, shape and also the size of the particles, as observed in Fig. 4b–d.

**Figure 5:**
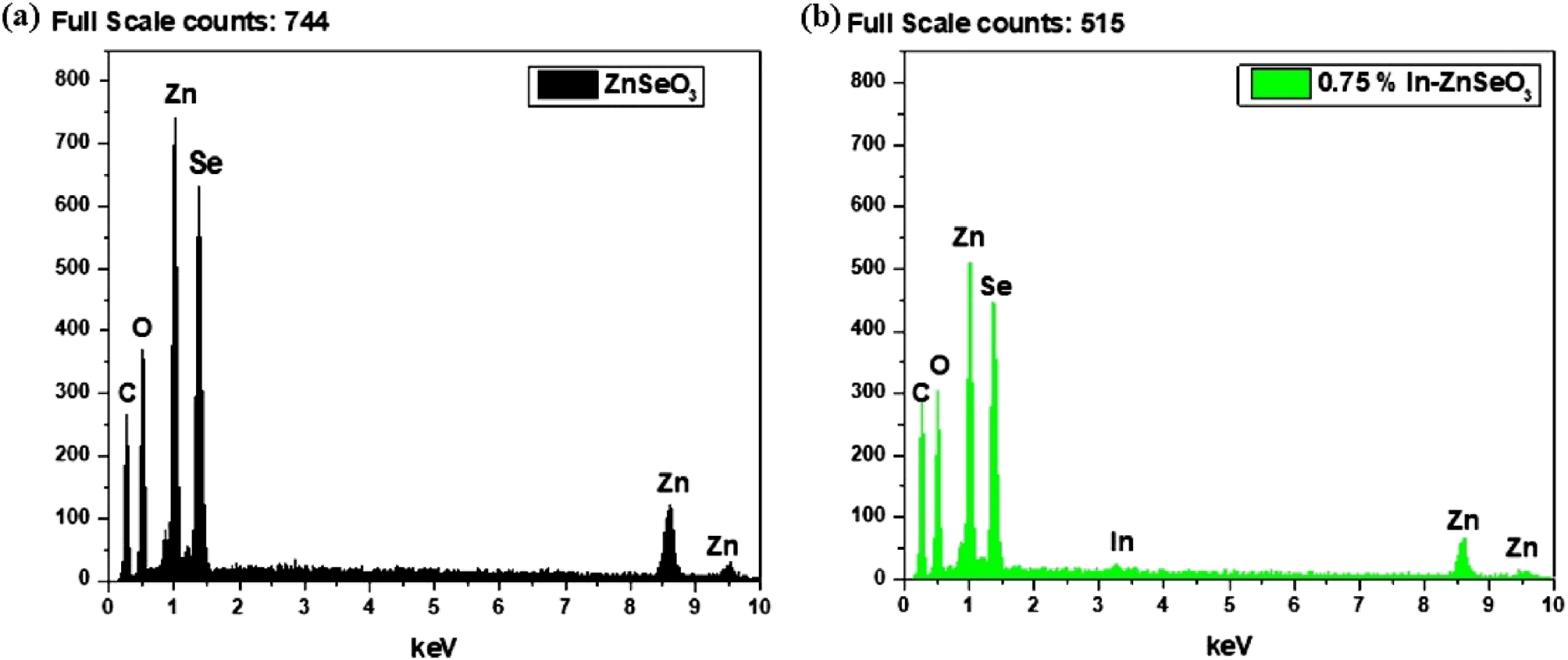
Electron dispersive spectroscopy of undoped-ZnSeO_3_ and 0.75% In- ZnSeO_3_nanopowder samples.

**Figure 6:**
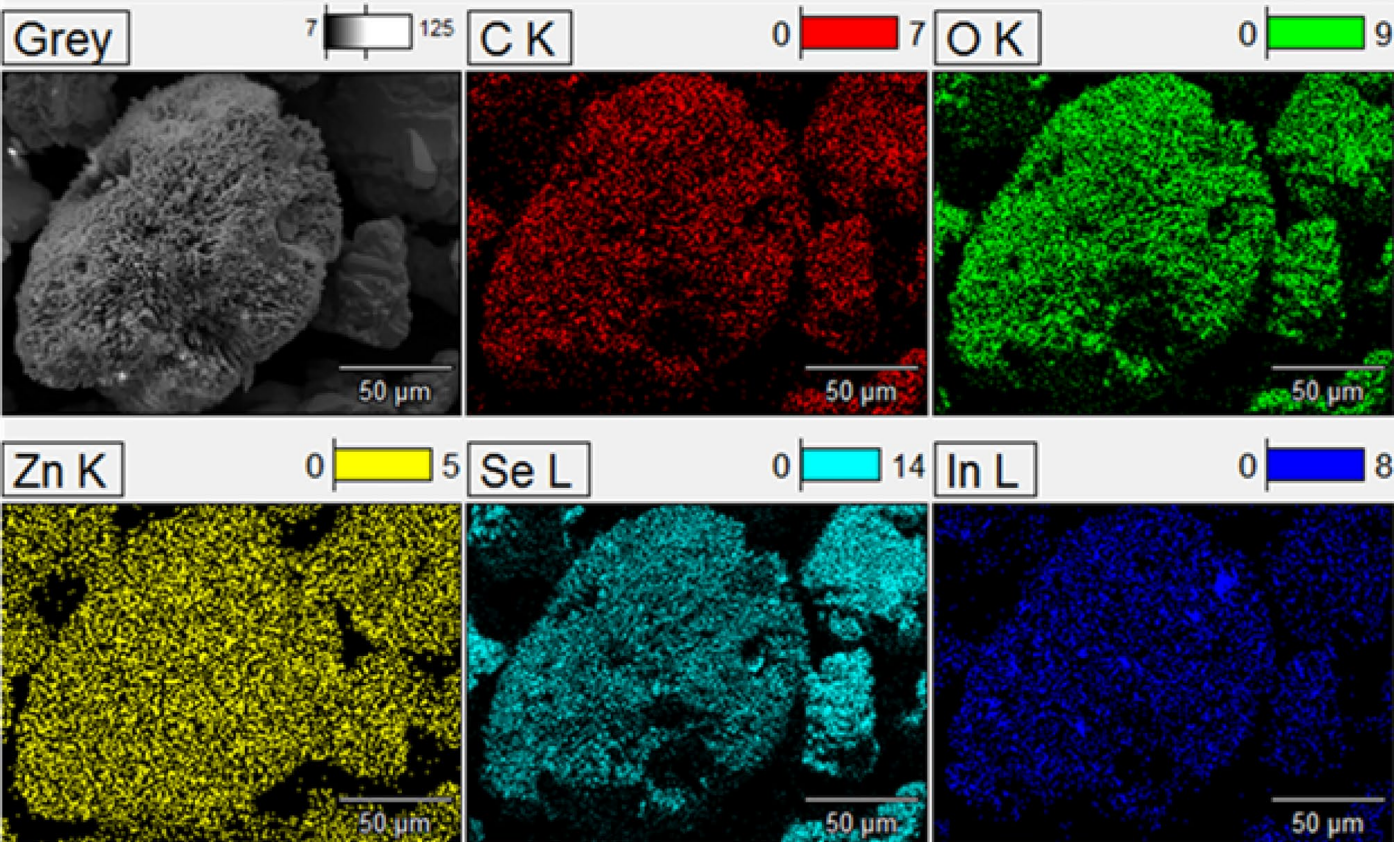
EDS mapping of 0.75% In–ZnSeO_3_ nanopowder sample.

Chemical composition of the elemental content for the synthesized ZnSeO_3_ nanopowder samples were measured using EDS spectroscopy. From Fig. 5a, it can be confirmed that the prepared sample is ZnSeO_3_ with no impurities observed. Figure 5b, indicates the presence of In and that In has successfully been doped into ZnSeO_3_ which supports the XRD data. Mapping was done to show the chemical distribution of the 0.75% In–ZnSeO_3_ as shown in Fig. 6. The distribution of all the other In doped samples are similar to that of the 0.75% In–ZnSeO_3_ hence, only one mapping has been shown.

### Raman spectroscopy

**Figure 7:**
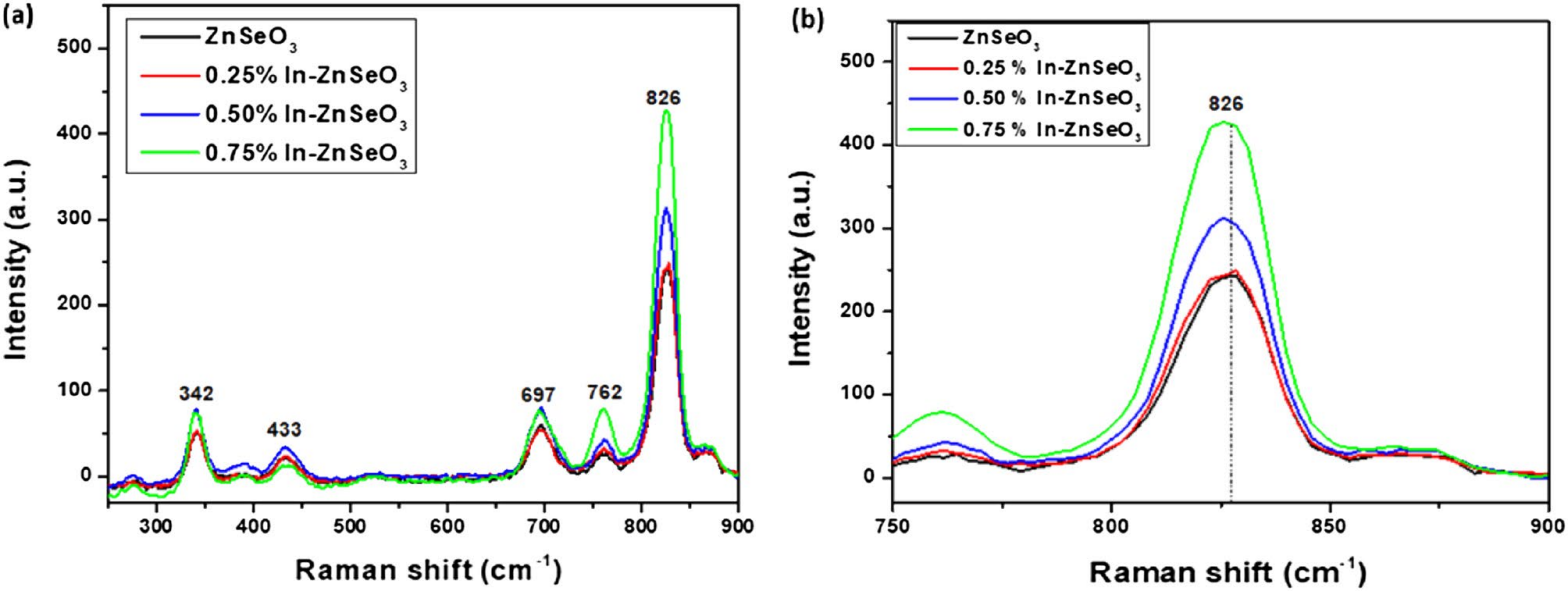
(**a**) Raman spectra, (**b**) Raman peak shiftof undoped ZnSeO_3_ and In-doped ZnSeO_3_ nanopowder samples.

Raman spectroscopy was performed to investigate the vibrational and electronic properties of the prepared undoped ZnSeO_3_ and In-doped ZnSeO_3_ nanopowder samples. To the best of our knowledge, there is not much Raman studies reported on ZnSeO_3_ hence in this study the Raman peaks have been associated with ZnO and ZnSe. Raman spectra of the undoped ZnSeO_3_ and In-doped ZnSeO_3_ samples are shown in Fig. 7a. There are Raman peaks at 342 (${E}_{2}^{high}-{E}_{2}^{low}$), 433 (${E}_{2}^{high}$), 697 (LO + TO), 762 (LO + TO) and 826 cm^-1^ (LO + TO) vibrational mode^27,28^ relating to ZnO. No vibrational modes are related to ZnSe separately as all these vibrational modes are related to ZnO which are because of the formation of ZnO occurring due to the higher temperatures that the samples are prepared in^29^.

If a crystal is exposed to a tensile stress, we can imagine the atoms being pulled apart, or chemical bonds length or the crystal structure being extended, comparative with their initial positions and lengths in an unstressed crystal^30^. As the bond length or lattice of the crystal increases, and the force constant remains the same, we ought to anticipate that the vibrational frequency should decrease^30^. This implies that if the crystal structure of a material is under tensile strain the shift is expected to move towards lower wavenumbers^30^. As it can be observed in Fig. 7b, the peaks are observed to be moving towards the lower wavenumbers, which indicates that the prepared samples are under tensile strain and these results are consistent with XRD results.

### UV–Vis spectroscopy

**Figure 8:**
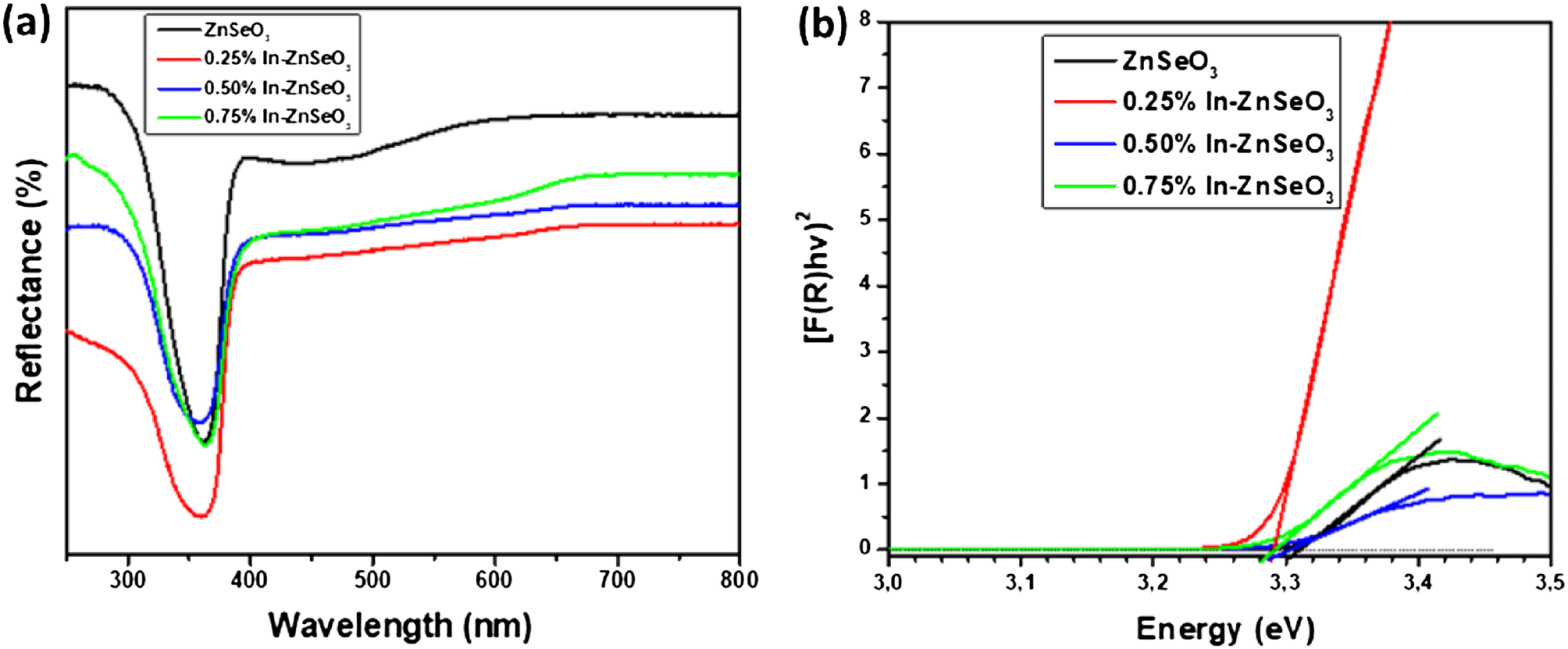
(**a**) UV–Vis spectra, (**b**) plot of [F(R) hν]^2^ versus hν of undoped ZnSeO_3_ and In-doped ZnSeO_3_nanopowder samples.

For studying the optical properties of the undoped ZnSeO_3_ and In-doped ZnSeO_3_ nanopowder samples, the nature of the band gap was determined utilizing the UV–Vis–NIR spectrophotometer in diffuse reflectance mode at room temperature over a scope of wavelength (250–800 nm). Figure 8a shows the diffuse reflectance spectra of the undoped ZnSeO_3_ and In-doped ZnSeO_3_ nanopowder samples. Diffuse reflectance spectra of all the samples revealed a characteristic absorption edge near 360 nm. Undoped ZnSeO_3_ sample revealed high reflectance in the visible region while the reflectance of the In-doped ZnSeO_3_ samples were observed to decrease. The energy band gap of the prepared samples were estimated using the Kubelka–Munk (K–M) function. This K–M theory describes the behaviour of the light path through a dispersing medium as a function of the scattering (S) and the absorption coefficients (k) with R_∞_ being the diffuse reflectance for an infinite sample^31^:

9 $$F\left({R}_{\infty }\right)=\frac{{\left(1-{R}_{\infty }\right)}^{2}}{2{R}_{\infty }}=\frac{k}{S}$$

In a parabolic band structure, the energy band gap and absorption coefficient are connected through the Tauc's Eq. ^32^.

10 $$\left(\alpha h\upsilon \right)=A{\left(h\upsilon -{E}_{g}\right)}^{m}$$

where α is the absorption coefficient of the sample, hν the photon energy, A is an energy autonomous constant, E_g_ is the energy band gap and m a consistent dependent upon the band gap nature by and generally m = 1/2 or 2 for allowed direct and indirect band gap material. Assuming the samples disperses in a completely diffuse way, K–M function can be written in the form of the Tauc's condition as^32^:

11 $$F\left({R}_{\infty }\right)h\upsilon =B{\left(h\upsilon -{E}_{g}\right)}^{m}$$

The energy band gaps for all prepared nanopowder samples were estimated from the plots of [F(R) hν]^2^ versus photon energy (hν) by extrapolation of the linear least square fit of [F(R) hν]^2^ to zero^31^. From Fig. 8b a red shift is observed in the energy band gap when indium is doped into ZnSeO_3_ nanopowder samples as it can be seen in Table 3. The red shift in the energy band gap may be attributed to the fact that In atoms have more radius than that of Zn and moreover the electronegativity of In is more than Zn^33^. These agrees with the structural properties that as Indium substitutes for Zn the structure of ZnSeO_3_ increases which tends to decrease the band gap energy^34^.

**Table 3:** Energy band gap.

Sample	E_g_ (eV)
ZnSeO_3_	3.3053
0.25% In–ZnSeO_3_	3.2761
0.50% In–ZnSeO_3_	3.2832
0.75% In–ZnSeO_3_	3.2775

### PL spectroscopy analysis

**Figure 9:**
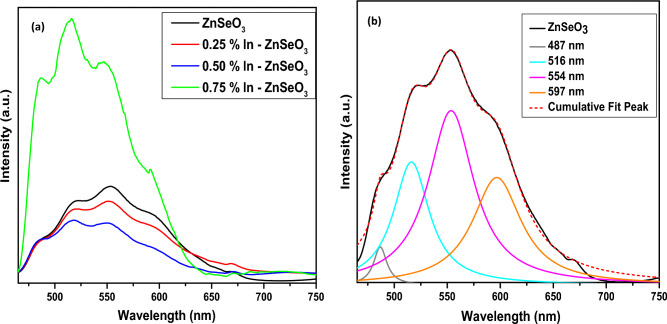
PL spectra of (**a**) undoped ZnSeO_3_ and In-doped ZnSeO_3_ nanopowder samples at different concentrations (**b**) Undoped ZnSeO_3_ deconvulated.

The photoluminescence (PL) spectra were recorded at room temperature in the range of 465–750 nm using an excitation wavelength of 340 nm. The photoluminescence (PL) measurements were conducted to study the effect of In concentrations on ZnSeO_3_. The PL spectrum of ZnSeO_3_ is characteristic of a combination of ZnO and ZnSe as less is known about ZnSeO_3_. Figure 9a presents the PL spectra of the undoped ZnSeO_3_ and In-doped ZnSeO_3_. The results show that PL emission intensity decreases from the undoped ZnSeO_3_ to 0.50% In–ZnSeO_3_ and then finally shows an increase in intensity for 0.75 In–ZnSeO_3_, which the phenomenon can be attributed to the increase of defect introduced by the dopant. All the In–doped ZnSeO_3_ samples indicated a shift towards lower wavelength as compared to the undoped ZnSeO_3_ sample indicating the presence of In in the ZnSeO_3_ structure. These observations also allows us to conclude that In has substituted Zn with Indium affecting the amount of defects taking part in the radiative recombination process^35^.

To acquire understanding into the emission, the PL emission spectra of undoped ZnSeO_3_ has been deconvulated as shown in Fig. 9b. The PL spectra show the four bands, with the peaks centred at around 487 nm, 516 nm, 554 nm and the other band at 597 nm. The blue emission band at around 487 nm credited to the radiative defects connected with the interface traps exciting at grain boundaries and produced from radiative transition between this level and the valence band^35,36^. Interestingly, the green emission peak around 516 nm is observed to increase with an increase in In concentration as the peak at 554 nm is seen to decrease. These may propose that the green emission band at 516 nm becoming dominant originates from In ions, which replaces zinc and consistently occurs in a small amount. Similar results were obtained by Thi Do and co-workers^37^ after doping ZnO with palladium. Ramya et al.^38^ attributed the peak around 554 nm to deep level trap emission, indicating that it might be originating from oxygen deficiency. The bands observed in the PL spectrum around 597 nm are like the orange PL emission bands observed in ZnSe. This band is assigned to the complicated centre comprising of a zinc vacancy and an impurity small donor: V_Zn_ + D^18^.

### TGA measurements

**Figure 10:**
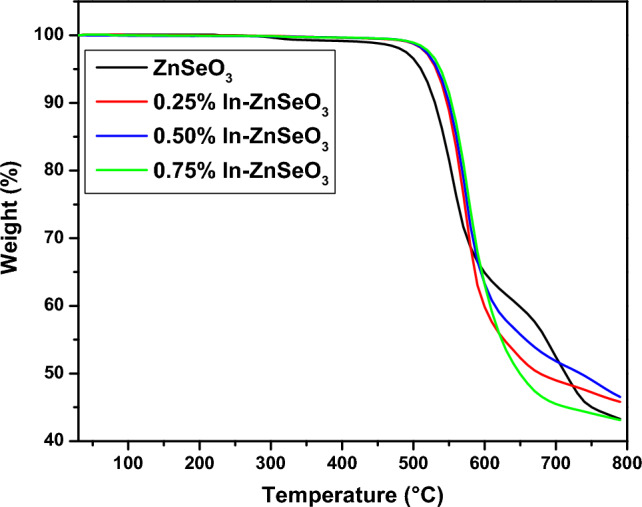
TGA of undoped ZnSeO_3_ and In-doped ZnSeO_3_nanopowder samples at different concentrations.

The Thermogravimetric analysis (TGA) was performed and represented in Fig. 10 to investigate the stability of the prepared samples as the temperature is increased. From Fig. 10, it can be observed that the undoped ZnSeO_3_ is the one that starts to have a weight lossquicker as compared to the In-doped ZnSeO_3_ nanopowder samples, with the 0.75% In–ZnSeO_3_ being the last one to start losing weight. Between 30 and 580 °C, the undoped ZnSeO_3_ is observed to have a weight loss of 31.44%, with 0.25% In–ZnSeO_3_, 0.50% In–ZnSeO_3_, and 0.75% In–ZnSeO_3_ having the weight loss of 31.26%, 28.23% and 25.88% respectively. This indicates that increasing the concentration of In improves the stability of ZnSeO_3_ against the mass loss as the temperature is increased as compared to when the ZnSeO_3_ is not doped. That is because as the dopant concentration is increased the prepared samples requires higher temperature in order to loss mass.

### Electrochemical impedance spectroscopy (EIS)

**Figure 11:**
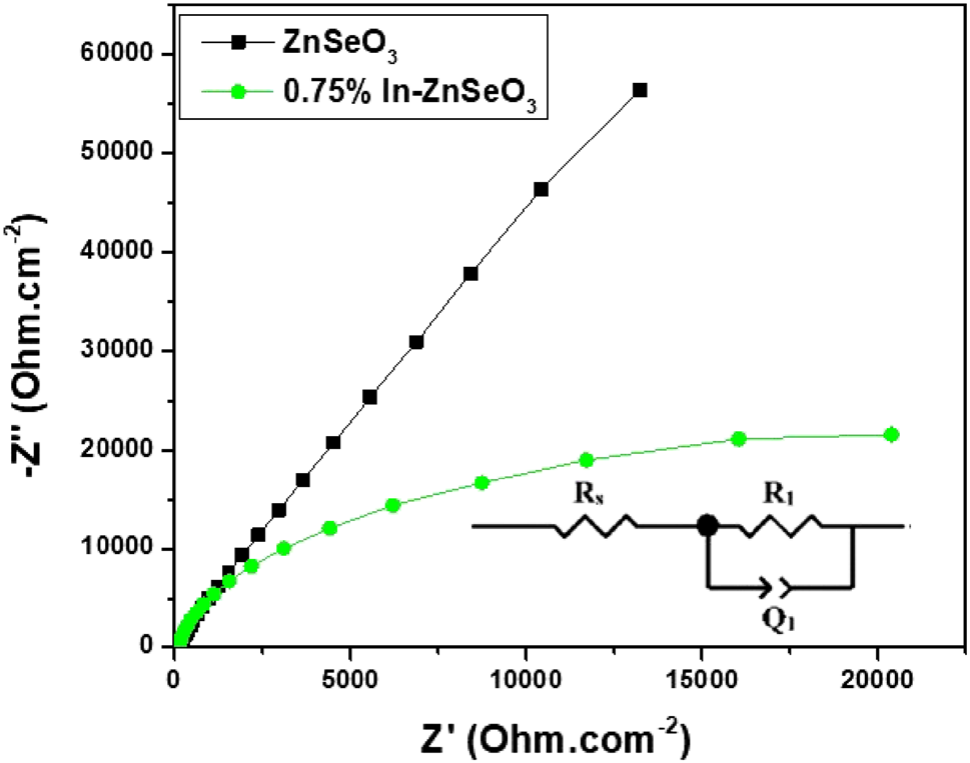
Nyquist plots of undoped ZnSeO_3_ and 0.75% In-doped ZnSeO_3_nanopowder samples under illumination condition.

To understand the charge mobility in the as-prepared thin films, electrochemical Impedance spectroscopy was conducted under open circuit conditions under 1 sun simulated solar radiation exposure. The results of Nyquist plots of the undoped and In-doped ZnSeO_3_ thin films prepared using an electrolyte containing 1 M Na_2_SO_4_ are shown in Fig. 11. Nyquist plots were fitted using the equivalent circuit displayed in the inset of Fig. 11, where, R_s_ is the series resistance, R_1_ is the charge transfer resistance and Q_1_ is the constant phase element. It can be observed from the Nyquist plot that the 0.75% In-doped ZnSeO_3_ the smaller semi-circle diameter as compared to the one of the undoped ZnSeO_3_ which suggests lower charge transfer resistance as the diameter of the semicircle is generally proportional to the resistance and flow of electrons at the interface^39^. The capacity (C) of the constant phase element was determined (see Table 4) from the equivalent circuit using the Eq. ^40^:

12 $$ C = \frac{{\left( {R_{1} Q_{1} } \right)^{1/2} }}{{R_{1} }} $$

**Table 4:** EIS parameters determined from the equivalent circuit.

Sample	R_s_	R_1_(kΩ)	Q_1_(µF)	n	C (µF)	Ref
BiVO_4_	58.80	0.53	81.00	0.80	–	^41^
Mo–BiVO_4_	53.00	0.43	115.70	0.80	–	
CoPi–BiVO_4_	58.10	0.27	122.80	0.80	–	
CoPi–Mo–BiVO_4_	51.40	0.18	108.10	0.80	–	
ZnSeO_3_	112.85	525.04	27.89	0.91	36.37	Present
0.75% In–ZnSeO_3_	87.45	21.95	47.32	0.93	47.46	Present

Interestingly, 0.75% In-doped ZnSeO_3_ thin film exhibited a higher chemical capacitance (C) as compared to undoped ZnSeO_3_, indicating higher electron density^35^. Hence, the lower the strain the higher the charge transport capabilities in photoanode.

## Conclusion

In the present study, undoped and In doped ZnSeO_3_ nanoparticles were successfully prepared by simple hydrothermal method. The structural, optical and Thermogravimetric studies were centred on the effect of dopants on the ZnSeO_3_ crystal structure. XRD, EDS and mapping results indicated that the prepared samples were of ZnSeO_3_ without any impurities present and that indium was succefully doped into ZnSeO_3_. Moreover, FE-SEM results showed that the prepared samples were orthorhombic corroborating the XRD results. In addition, the increase of In concentration caused an expansion on the ZnSeO_3_ crystal structure (from 537.948 to 541.079 Å^3^) indicating the presence of tensile strain and these results were supported by the Raman results which also indicated a Raman shift towards lower wavenumbers. The UV–vis measurements indicated a reduction in the energy band gap from 3.305 to 3.278 eV with increase of In concentration indicating that In–ZnSeO_3_ can also absorb energy at lower wavelength. Furthermore, the PL spectra was observed to show the blue and green emissions with the green emission indicating the presence of defects related to In as it replaced Zn in the crystal lattice. On top of that, EIS measurements revealed that the 0.75% In-doped ZnSeO_3_ thin film had better charge transport behaviour as compared to the undoped ZnSeO_3_. These has demonstrated the effect of strain engineering in the ZnSeO_3_ nanoparticles also indicating that In-doped ZnSeO_3_ is a promising candidate for photoanode applications.

## Data Availability

The datasets used and/or analysed during the current study is available from the corresponding author on reasonable request.

## References

[1] Taya SA (2013). Dye-sensitized solar cells using fresh and dried natural dyes. Int. J. Mater. Sci. Appl..

[2] Sumathi T, Fredrick SR, Deepa G, Shkir M, Hakami J, Umar A (2022). Construction of nickel molybdenum sulfide (NiMoS_3_)/bio carbon (BC) heterostructure photoanodes and optimization of light scattering to improve the photovoltaic performance of dye sensitized solar cells (DSSCs). Phys. E Low Dimens. Syst. Nanostructures.

[3] Aneesiya KR, Louis C (2020). Localized surface plasmon resonance of Cu-doped ZnO nanostructures and the material’s integration in dye sensitized solar cells (DSSCs) enabling high open-circuit potentials. J. Alloys Compd..

[4] Shaikh SMF, Rahman G, Mane RS, Joo OS (2013). Bismuth oxide nanoplates-based efficient DSSCs: Influence of ZnO surface passivation layer. Electrochim. Acta.

[5] Dou X, Prabhakar RR, Mathews N, Lam YM, Mhaisalkar S (2012). Zn-doped SnO_2_ nanocrystals as efficient DSSC photoanode material and remarkable photocurrent enhancement by interface modification. J. Electrochem. Soc..

[6] Dou X, Sabba D, Mathews N, Wong LH, Lam YM, Mhaisalkar S (2011). Hydrothermal synthesis of high electron mobility Zn-doped SnO_2_ nanoflowers as photoanode material for efficient dye-sensitized solar cells. Chem. Mater..

[7] Deng S, Sumant AV, Berry V (2018). Strain engineering in two-dimensional nanomaterials beyond graphene. Nano Today.

[8] Feng J, Qian X, Huang CW, Li J (2012). Strain-engineered artificial atom as a broad-spectrum solar energy funnel. Nat. Photonics.

[9] Jin L, Wang Y, Wu J, Su C, Zhou H, Xu H (2022). Properties of oxidation quantum dots-CdO/TiO_2_ heterostructures constructed as DSSC photoanodes. Mater. Sci. Semicond. Process..

[10] Moorthy S, Moorthy G, Swaminathan K (2020). Fabrication of novel ZnSeO_3_ anchored on g-C_3_N_4_ nanosheets: An outstanding photocatalyst for the mitigation of pesticides and pharmaceuticals. J. Inorg. Organomet. Polym. Mater..

[11] Ghos BC (2021). Influence of the substrate, process conditions, and postannealing temperature on the properties of ZnO thin films grown by the successive ionic layer adsorption and reaction method. ACS Omega.

[12] Rama Krishna C, Kang M (2017). Improving the photovoltaic conversion efficiency of ZnO based dye sensitized solar cells by indium doping. J. Alloys Compd..

[13] Rini AS, Rati Y, Agustin M, Hamzah Y, Umar AA (2020). Seed-mediated synthesis and photoelectric properties of selenium doped zinc oxide nanorods. Sains Malays..

[14] Esakki ES, Vivek P, Devi LR, Sarathi R, Sheeba NL, Sundar SM (2022). Influence on electrochemical impedance and photovoltaic performance of natural DSSC using *Terminalia*
*catappa* based on Mg-doped ZnO photoanode. J. Indian Chem. Soc..

[15] Luo J, Wang Y, Zhang Q (2018). Progress in perovskite solar cells based on ZnO nanostructures. Sol. Energy.

[16] Jabri S (2016). Study of the optical properties and structure of ZnSe/ZnO thin films grown by MOCVD with varying thicknesses. Phys. B Condens. Matter.

[17] Ghoul M (2015). Synthesis of core/shell ZnO/ZnSe nanowires using novel low cost two-steps electrochemical deposition technique. J. Alloys Compd..

[18] Dauletbekova A (2022). Ion-track template synthesis and characterization of ZnSeO_3_ nanocrystals. Crystals.

[19] Hassan SA, Bashir S, Zehra K, Ahmed QS (2018). Structural, morphological and optical properties of pulsed laser deposited ZnSe/ZnSeO_3_ thin films. Mater. Res. Express.

[20] Gan YX, Jayatissa AH, Yu Z, Chen X, Li M (2020). Hydrothermal synthesis of nanomaterials. J. Nanomater..

[21] Maswanganye MW, Kabongo GL, Dhlamini MS (2023). Modulating charge mobility in microwave synthesized Ti-doped ZnS nanoparticles for potential photoanode applications. Nanomaterials.

[22] Xia Z, Guo S (2019). Strain engineering of metal-based nanomaterials for energy electrocatalysis. Chem. Soc. Rev..

[23] Hawthorne FC, Ercit TS, Groat LA (1986). Structures of zinc selenite and copper selenite. Acta Crystallogr. Sect. C Cryst. Struct. Commun..

[24] Norouzzadeh P, Mabhouti K, Golzan MM, Naderali R (2020). Consequence of Mn and Ni doping on structural, optical and magnetic characteristics of ZnO nanopowders: The Williamson–Hall method, the Kramers–Kronig approach and magnetic interactions. Appl. Phys. A Mater. Sci. Process..

[25] Al-Ariki S, Yahya NAA, Al-A’nsi SA, Jumali MHH, Jannah AN, Abd-Shukor R (2021). Synthesis and comparative study on the structural and optical properties of ZnO doped with Ni and Ag nanopowders fabricated by sol gel technique. Sci. Rep..

[26] Munawar T, Shahid M, Faisal N, Muhammad M (2022). Transition metal-doped SnO_2_ and graphene oxide (GO) supported nanocomposites as efficient photocatalysts and antibacterial agents. Environ. Sci. Pollut. Res..

[27] Verma M, Kaswan A, Patidar D, Sharma KB, Saxena NS (2016). Phase transformation and thermal stability of ZnSe QDs due to annealing: Emergence of ZnO. J. Mater. Sci. Mater. Electron..

[28] Šćepanović M, Grujić-Brojčin M, Vojisavljević K, Bernikc S, Srećković T (2010). Raman study of structural disorder in ZnO nanopowders. J. Raman Spectrosc..

[29] Cuscó R (2007). Temperature dependence of Raman scattering in ZnO. Phys. Rev. B Condens. Matter. Mater. Phys..

[30] Tuschel D (2019). Stress, strain, and Raman spectroscopy. Spectroscopy (Santa Monica).

[31] Gesesse GD, Gomis-Berenguer A, Barthe MF, Ania CO (2020). On the analysis of diffuse reflectance measurements to estimate the optical properties of amorphous porous carbons and semiconductor/carbon catalysts. J. Photochem. Photobiol. A Chem..

[32] Kalu O, Duarte Moller JA, Reyes Rojas A (2019). Structural and optical properties of cadmium magnesium zinc oxide (CdMgZnO) nanoparticles synthesized by sol–gel method. Phys. Lett. Sect. A Gen. At. Solid State Phys..

[33] Patil SJ, Lokhande VC, Lee DW, Lokhande CD (2016). Electrochemical impedance analysis of spray deposited CZTS thin film: Effect of Se introduction. Opt. Mater. (Amst).

[34] Reshak AH (2014). Structural, electronic and optical properties in earth-abundant photovoltaic absorber of Cu_2_ZnSnS_4_ and Cu_2_ZnSnSe_4_ from DFT calculations. Int. J. Electrochem. Sci..

[35] Arora D, Asokan K, Mahajan A, Kaur H, Singh DP (2016). Structural, optical and magnetic properties of Sm doped ZnO at dilute concentrations. RSC Adv..

[36] Jin BJ, Im S, Lee SY (2000). Violet and UV luminescence emitted from ZnO thin films grown on sapphire by pulsed laser deposition. Thin Solid Films.

[37] Thi Do AT, Giang HT, Thi Do T, Pham NQ, Ho GT (2014). Effects of palladium on the optical and hydrogen sensing characteristics of Pd-doped ZnO nanoparticles. Beilstein J. Nanotechnol..

[38] Ramya E, Rao MV, Jyothi L, Rao DN (2018). Photoluminescence and nonlinear optical properties of transition metal (Ag, Ni, Mn) doped ZnO nanoparticles. J. Nanosci. Nanotechnol..

[39] Munawar T (2022). Facile synthesis of rare earth metal dual-doped Pr_2_O_3_ nanostructures: Enhanced electrochemical water-splitting and antimicrobial properties. Ceram. Int..

[40] Azmand A, Kafashan H (2019). Al-doped ZnS thin films: Physical and electrochemical characterizations. J. Alloys Compd..

[41] Liu C (2019). Cobalt–phosphate-modified Mo:BiVO_4_ mesoporous photoelectrodes for enhanced photoelectrochemical water splitting. J. Mater. Sci..

