# Author Correction: Unveiling the effect of strain engineering on the electrochemical properties of hydrothermally grown nanostructured indium doped ZnSeO_3_ for photoanode applications

**DOI:** 10.1038/s41598-023-50647-7

**Published:** 2024-01-11

**Authors:** M. W. Maswanganye, G. L. Kabongo, L. E. Mathevula, B. M. Mothudi, M. S. Dhlamini

**Affiliations:** 1 Department of Physics, University of South Africa, Florida Park, Roodepoort, 1709 Republic of South Africa; 2 Department of Physics, University of Limpopo, Private Bag X1106, Sovenga, 0727 South Africa

Correction to: *Scientific Reports* 10.1038/s41598-023-47436-7, published online 16 November 2023

The original version of this Article omitted an affiliation for M. W. Maswanganye. The correct affiliations are listed below.

Department of Physics, University of South Africa, Florida Park, Roodepoort 1709, Republic of South Africa.

Department of Physics, University of Limpopo, Private Bag X1106, Sovenga, 0727, South Africa.

The original Article has been corrected.

